# 66361 TL1 Team Approach to Predicting Response to Spinal Cord Stimulation for Chronic Low Back Pain

**DOI:** 10.1017/cts.2021.685

**Published:** 2021-03-30

**Authors:** Kyle See, Rachel Ho, Stephen Coombes, Ruogu Fang

**Affiliations:** 1 University of Florida Biomedical Engineering; 2 University of Florida Applied Physiology and Kinesiology

## Abstract

ABSTRACT IMPACT: Understanding how spinal cord stimulation works and who it works best for will improve clinical trial efficacy and prevent unnecessary surgeries. OBJECTIVES/GOALS: Spinal cord stimulation (SCS) is an intervention for chronic low back pain where standard interventions fail to provide relief. However, estimates suggest only 58% of patients achieve at least 50% reduction in their pain. There is no non-invasive method for predicting relief provided by SCS. We hypothesize neural activity in the brain can fill this gap. METHODS/STUDY POPULATION: We tested SCS patients at 3 times points: baseline (pre-surgery), at day 7 during the trial period (post-trial), and 6 months after a permanent system had been implanted. At each time point participants completed 10 minutes of eyes closed, resting electroencephalography (EEG) and self-reported their pain. EEG was collected with the ActiveTwo system and a 128-electrode cap. Patients were grouped based on the percentage change of their pain from baseline to the final visit using a median split (super responders > average responders). Spectral density powerbands were extracted from resting EEG to use as input features for machine learning analyses. We used support vector machines to predict response to SCS. RESULTS/ANTICIPATED RESULTS: Baseline and post-trial EEG data predicted SCS response at 6-months with 95.56% and 100% accuracy, respectively. The gamma band had the highest performance in differentiating responders. Post-trial EEG data best differentiated the groups with feature weighted dipoles being more highly localized in sensorimotor cortex. DISCUSSION/SIGNIFICANCE OF FINDINGS: Understanding how SCS works and who it works best for is the long-term objective of our collaborative research program. These data provide an important first step towards this goal.

